# Amyloid cast tubulopathy: a unique form of immunoglobulin-induced renal disease

**DOI:** 10.1038/bcj.2016.74

**Published:** 2016-09-23

**Authors:** I-A Iliuta, A P Garneau, E Latulippe, P Isenring

**Affiliations:** 1 Department of Nephrology, Québec, QC, Canada; 2 Department of Medicine, Pathology Group, L'Hôtel-Dieu du CHU de Québec, Laval University, Québec, QC, Canada

Excess production of monoclonal immunoglobulins (Ig) occurs in ~1% of all individuals beyond the age of 50.^1^ The overall prevalence of kidney disease in this condition, that is, of Ig-induced kidney disease (Ig-KD) is over 20%, and its presence is associated with a wide variety of nephropathological lesions (Table 1) as well as underlying pathogenic mechanisms.

The most classic Ig-KD is cast nephropathy, also called myeloma kidney. It corresponds to an Ig-induced tubular disease (Ig-TD) that generally occurs in the setting of multiple myeloma. The other types of lesions, however, are associated with a wider variety of plasma cell dyscrasias including monoclonal gammopathy of undetermined significance.

On vary rare occasions, Ig-TD is limited to the proximal nephron. Under such circumstances, the lesion usually corresponds to cytosolic κ1-restricted crystalline deposits or to λ-restricted lysosomal abnormalities.^1, 2^ On the basis of a study by Larsen *et al.*,^2^ the latter entity could be underdiagnosed as it corresponds to a more subtle renal injury in which lysosomes are typically increased in number and mottled in texture. Cytosolic fibrillary deposits in the absence of crystal formation have also been identified in a few cases.^3, 4, 5, 6^

In the current report, we describe an even rarer, if not unique, form of Ig-TD. This entity was identified in a 54-year-old diabetic man admitted for renal failure (creatinine 5.49 mg/dl) and found to have an IgG λ gammopathy due to multiple myeloma in the absence of Fanconi syndrome. As seen in Figure 1, the lesion consisted of numerous spherical deposits that were confined to the cytoplasm of the proximal nephron (upward arrows) and to the lumen of many nephron segments where they formed aggregates (downward arrows). The deposits also shared a unique combination of features in that they were pale by hematoxylin and eosin, and periodic acid-Schiff (PAS) (a and b), positive by trichrome and Congo red (c and d), fibrillary (e and f) and λ-restricted (not shown).

From these findings, we concluded that the pathological picture observed belonged to the highly uncommon category of isolated fibrillary Ig-TD of which a subset is termed amyloid tubulopathy (AT) in the presence of Congo red-positive deposits. Such an entity has only been reported twice thus far.^5, 6^ In the current case, surprisingly, it was also accompanied by amyloid casts (AC), a type of lesion that has already been reported^6, 7, 8, 9, 10^ but that was described previously as spiculated structures within the periphery of atypical casts—instead of intraluminal nodular structures—and that was generally identified in the absence of AT. The other subset of isolated fibrillary Ig-TD is Congo red-negative, is also quite uncommon^3, 4^ and could correspond to a variant of fibrillary or immunotactoid glomerulopathy.

At times, plasma cell dyscrasias can induce two types of renal lesions in the same individual. In our patient, AC and AT could have still resulted from a single process in which Ig β-fibrillary structures formed in the urinary space were endocytosed by proximal tubular cells. This hypothesis would be consistent with the absence of amyloid deposition in other renal structures and the limited ability of β-fibrils to permeate the glomerular filtration barrier. It would also suggest that AT, AC and AC-associated AT all correspond to distinct entities.

On the basis of our observations, it appears that isolated Ig-TD could now come into as much as nine different entities, expanding further the list of lesions that can develop in the face of excess monoclonal Ig production. In Table 1, we have regrouped these different entities based on the type of Ig deposits formed, that is, fibrillary, crystalloid or plain Ig deposits, and are using the term amyloid cast tubulopathy (ACT) to designate the new form of Ig-TD identified through this case.

## Figures and Tables

**Figure 1:**
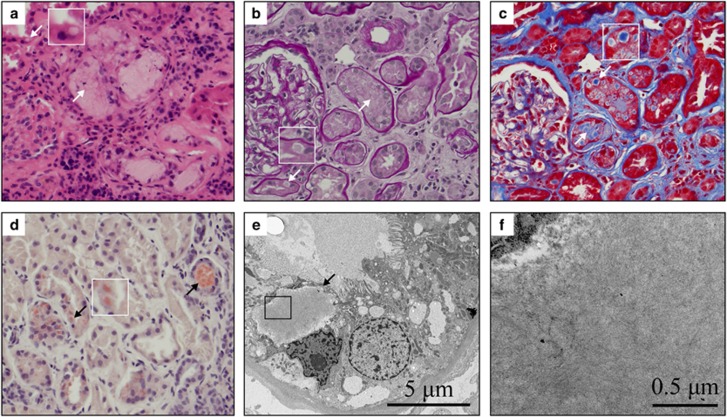
Histological and ultrastructural characteristics of renal lesions in the current case. (**a**) Hematoxylin and eosin. (**b**) Periodic acid-Schiff. (**c**) Trichrome. (**d**) Congo red. Isolated cytosolic deposits are shown by arrows pointing downward and through insets in which they are magnified further. Clumped intraluminal deposits are shown by arrows pointing upward. A dense plasma cell infiltrate is also seen in **a**. (**e** and **f**) Electron microscopy. Micrographs were taken at × 5000- and × 40 000-magnification, respectively). The box in **e** represents the field that was magnified in **f**. Amyloid fibrils measured ~8 nm in diameter.

**Table 1:** Types of Ig-KD

*Ig-GD (with or without tubulointerstitial deposition)*
Fibrillary Ig
Amyloidosic (L, HC, LCHC)
Predominantly vascular
Predominantly glomerular
Non-Amyloidosic
Immunotactoid (LCHC)
With glomerulonephritis (LCHC)
With cryoglobulinemia type I (LCHC)
Fibrillary *per se* (LCHC)

Crystalloid Ig
With cryoglobulinemia type I
Crystal-storing histiocytosis

Plain (Ig or C)
With glomerulosclerosis (LC, HC, LCHC)
With glomerulonephritis
Ig glomerulonephritis (LCHC)
C3 glomerulonephritis (C3)
C4 glomerulonephritis (C4)
*Isolated Ig-TD*
Fibrillary Ig
Amyloidosic
Amyloid tubulopathy
Amyloid cast
Amyloid cast tubulopathy
Interstitial
Non-Amyloidosic: immunotactoid or fibrillary?

Crystalloid Ig
Plain Ig
Cast nephropathy
Lysosomal
Interstitial fibrosis with our without interstitial nephritis

Abbreviations: HC, heavy chain; LC, light chain. Deposits can also be found in the tubulointerstitial compartment.

In crystalline Ig-TD, deposits occur in the lumen, cytosol and lysosomes of proximal tubular cells, are negative by PAS and birefringent. In non-amyloidosic Ig-TD, deposits occur in the cytosol, are spherical, contain tightly joined fibrils of ~8 nm diameter and lead to cellular vacuolization. Crystalline and lysosomal Ig-TD are often associated with Fanconi syndrome.
